# The Editor's Letter-Box

**Published:** 1921-05-14

**Authors:** Gilbert G. Panter

**Affiliations:** Secretary. Great Northern Central Hospital, Holloway Road, N.


### THE EDITOR'S LETTER-BOX.

### IS A HOSPITAL PAGEANT PRACTICABLE?

*To the Editor of* THE HOSPITAL.

SIR,—I have read your proposals about "A Hospital Pageant" in THE HOSPITAL of the 7th inst. with very great interest, and I am quite in agreement with the practical suggestion made. I feel that at the present time hospitals should combine in securing the utmost publicity, as the general public has few opportunities of learning of the valuable work done by these institutions. The difficulties confronting all hospitals are so great that it is only by joint effort and propaganda that they will be surmounted, and I hope it will be possible to carry out the idea of a Pageant to precede and help Hospital Sunday.

The Press always gives generous support to hospitals, and doubtless all the newspapers would lend their aid in bringing the Pageant to the notice of the public, thus helping to swell the collections for Hospital Sunday, while the indirect benefit received by all institutions would be inestimable. I will assist the movement in any way possible.—Yours faithfully,

GILBERT G. PANTER, Secretary.

Great Northern Central Hospital, Holloway Road, N.

